# Preferences for implementing training program among primary care physicians in prescribing and deprescribing for patients with multimorbidity: a discrete choice experiment

**DOI:** 10.3389/fmed.2026.1795722

**Published:** 2026-03-13

**Authors:** Leyi Jiang, Yu Xia, Liyan Han, Ming Yan, Xinmei Zhou, Ruoxia Zhang, Yiting Lu, Jiaying Yu, Guifen Zhang, Lingyan Wu, Yi Guo, Yuling Tong, Zhijie Xu

**Affiliations:** 1 Department of General Practice, The Second Affiliated Hospital, Zhejiang University School of Medicine, Hangzhou, Zhejiang, China; 2 Department of General Practice, Peking University Shenzhen Hospital, Shenzhen, China; 3 Zhangqi Township Health Center of Cixi City, Ningbo, China; 4 Department of Emergency Medicine, Sir Run Run Shaw Hospital, Zhejiang University School of Medicine, Hangzhou, China; 5 Zhongdai Street Community Health Service Center, Pinghu, China

**Keywords:** deprescribing, discrete choice experiment, multimorbidity, prescribing, primary care physicians

## Abstract

**Background:**

Primary care physicians (PCPs) have great needs of receiving training and potential to improve their capacities of prescribing and deprescribing for patients with multimorbidity. However, limited studies focused on PCPs’ preferences for how training program is implemented. This study used a discrete choice experiment (DCE) to investigate PCPs’ preferences for implementing training program in prescribing and deprescribing for patients with multimorbidity.

**Methods:**

This study surveyed PCPs in eastern China from October 10 to December 5, 2024 using an electronic questionnaire. Eight attributes and levels in a DCE were identified based on implementation science theory: instructor composition, teaching model, training location, participant enrollment, session duration, training frequency, assessment methods, and theoretical basis of the course. DCE data were analyzed using a mixed logit model to explore respondents’ attribute preferences. Relative importance scores and predicted changes in training implementation selection rates were calculated.

**Results:**

The preference model included 2,976 choice observations from 248 respondents. These respondents were 50.4% male and the overall average age was 37.7 years. They showed significant preferences for five attributes, ranked in order of relative importance score: training frequency (37.29%), session duration (27.23%), instructor composition (16.29%), participant enrollment (10.23%), and theoretical basis of the course (8.95%). Scenario analysis revealed that optimizing high-preference attributes (e.g., shorter duration, multidisciplinary instructors) could increase acceptance of weekly training by up to 66.75%.

**Conclusion:**

PCPs prioritize time-efficient, high-quality training featuring monthly 45-min sessions, autonomous enrollment, multidisciplinary mentoring, and the use of clinical practice guidelines. Autonomy and flexible formats may increase PCPs’ participation, and a modular, interdisciplinary and demand-driven approach is needed to optimize prescribing competencies in multimorbidity care.

## Introduction

Primary care physicians (PCPs) serve as key gatekeepers within the healthcare system, particularly in the management of patients with multimorbidity through the provision of continuous, coordinated, and accessible care. The quality of their prescribing practices directly influences patient safety and the efficient use of healthcare resources. However, evidence suggests that the prevalence of potentially inappropriate medications among older adults in primary care settings in China is as high as 39% (1). PCPs continue to face substantial challenges in both prescribing and deprescribing medications for patients with multimorbidity (2). Clinical decision-making is often complicated by factors such as drug–drug interactions, cognitive biases, and treatment burden. Additional barriers include patient resistance to changes in existing treatment regimens, limited access to continuing medical education, and time constraints during patient encounters (3).

These challenges are compounded by the escalating burden of multimorbidity worldwide. As chronic diseases become more prevalent and populations continue to age, the presence of multiple chronic conditions within individuals has emerged as a critical public health challenge (4–6). In China, the prevalence of multimorbidity among older adults has demonstrated an increasing trend over recent years, rising to 43% in 2023 (7). Among individuals with multimorbidity, polypharmacy and inappropriate medication use are widespread concerns (1). An increase in the number of prescribed medications is associated with a higher risk of drug–drug interactions, drug-disease interactions, and medication errors, collectively termed medication-related problems, which pose substantial threats to patient safety with consequences ranging from functional decline to mortality (8–10).

To address these challenges, the Chinese government has introduced a series of policies aimed at enhancing the quality of medical services as part of ongoing healthcare reforms (11). Among these initiatives, promoting rational drug use through professional training has been identified as a key strategy. Prior research indicates that well-designed training programs can significantly enhance prescribing accuracy and reduce the incidence of potentially inappropriate prescriptions (12, 13). However, training effectiveness depends not only on content but also on implementation formats that influence knowledge retention and participant engagement (14, 15). Accordingly, a comprehensive understanding of PCPs’ training preferences is essential for the development of a practical, effective, and sustainable competency-based training system.

Understanding PCPs’ implementation preferences is a prerequisite for designing interventions that achieve intended outcomes (16). Even expertly designed training content fails to improve practice if physicians do not participate or engage. From a theoretical perspective, training effectiveness depends on a causal chain in which implementation attributes influence participation decisions, participation enables knowledge acquisition, and knowledge translates into practice change (17). Within this chain, individual preferences function as the initial gateway determining whether training can exert any effect.

Equipped with concepts and tools from implementation science, this challenge can be systematically addressed. Defined as “the scientific study of methods to promote the systematic uptake of research findings and other evidence-based practices into routine practice” (18), implementation science offers theoretical frameworks and practical tools to identify and overcome barriers to implementation, adapt interventions to specific healthcare settings, and promote sustainable practice change (19). Rather than merely identifying what content PCPs wish to learn, we deconstruct training programs into discrete implementable attributes informed by implementation strategy frameworks. This approach enables quantification of preferences for specific design features and generates evidence for precise matching of implementation strategies to PCPs’ priorities (20). The significance therefore lies not only in describing preferences but in providing an empirical basis for designing theory-driven, contextually tailored interventions that can be systematically optimized and replicated.

However, research exploring PCPs’ preferences for how training programs should be implemented, particularly in prescribing and deprescribing for multimorbidity, remains limited. Existing studies have concentrated on content design and instructional method evaluation, with comparatively little attention to implementation preferences (13, 15, 21). This study utilizes a discrete choice experiment (DCE) to explore PCPs’ preferences regarding the implementation of training programs in prescribing and deprescribing for patients with multimorbidity. DCE is a multidimensional stated preference method that allows for the identification and quantification of participants’ trade-offs among competing attributes (22). DCE has been used to assess decision-making in areas ranging from disease screening to preferences for workforce incentives (23–25). By identifying the factors influencing PCPs’ preferences, this study aims to align training initiatives closely with their needs, potentially enhancing engagement and improving the feasibility and effectiveness of the programs.

## Methods

This study employed a DCE to systematically investigate PCPs’ preferences regarding the implementation of training programs. DCE is particularly well-suited for this purpose, as it enables the quantification of the relative importance of multiple attributes by presenting respondents with realistic scenarios that require trade-offs between competing options (22). This multi-attribute decision-making framework aligns with the study’s objectives and supports the identification of optimal training configurations (26). Compared with traditional survey methods, which may struggle to capture the complexity of individual preferences with sufficient reliability and validity (27, 28), DCE provides a more robust and scientifically rigorous approach. In particular, the use of a mixed logit model facilitates accurate estimation of utility values while accounting for heterogeneity in respondent preferences across different training attributes.

This study involves human participants and was approved by the Second Affiliated Hospital of Zhejiang University School of Medicine Ethics Committee [approval no. (2024) Ethics Review Research no. (1485)], and the research was conducted in accordance with the principles outlined in the Declaration of Helsinki.

### Study settings and population

Participants were recruited from 23 community health centers located in Hangzhou, Cixi, Daishan, Suichang, and Kaihua. Inclusion criteria were as follows: (a) licensed clinicians whose scope of practice includes general practice or internal medicine, and (b) those who had been employed at a community health center for at least 1 year. Physicians were excluded if they declined to provide informed consent or if they had no intention of participating in any future training programs.

Sample size estimation in this study utilized the established rule-of-thumb formula developed by Johnson and Orme (29): *n* > 500*c*/(*t* × *a*), where *n* represents the minimum required sample size, *c* denotes the largest number of levels across all attributes, *t* indicates the number of choice sets presented to each respondent, and *a* signifies the number of alternatives per choice set. Application of this formula yielded a minimum required sample size of $N>\frac{500\times 2}{6\times 2}=83$ respondents per questionnaire version. With two questionnaire versions implemented, the aggregate minimum sample size was determined to be 166 respondents. To compensate for anticipated invalid or incomplete responses (estimated at 20%), the target sample size underwent appropriate adjustment, establishing a final recruitment objective of at least 208 PCPs meeting eligibility criteria.

### The design of DCE

#### Attributes and levels

The identification and specification of training attributes and their corresponding levels represent the foundational and most critical step in designing a DCE, as they play a pivotal role in uncovering the factors that influence training preferences. To comprehensively identify the attributes relevant to PCPs’ preferences for implementing the training program, this study employed a multi-step process, including a systematic review of the literature, structured group discussions, and qualitative interviews.

Based on the relevant literatures (28, 30–32), an initial candidate pool was formed, comprising instructor composition, teaching model, training location, participant enrollment, session duration, training frequency, assessment methods, theoretical basis of the course, training depth, training scale, accreditation type, and incentive mechanisms. Secondly, a panel of eight experts comprising primary care physicians, clinical pharmacists, medical educators, and implementation science researchers evaluated these candidates in structured discussions. Attributes were prioritized based on their potential influence on physician participation and alignment with implementation science frameworks (33). Training depth, training scale, accreditation type, and incentive mechanisms were removed due to limited variability or lower relevance, yielding a preliminary set of eight attributes. Thirdly, semi-structured interviews with 20 primary care physicians recruited from two community health centers confirmed the relevance of these attributes; feedback led to simplified wording for participant enrollment and theoretical basis of the course, with no attributes removed.

Following attribute identification, the corresponding levels were determined. For quantitative attributes including session duration and training frequency, levels were selected to reflect common continuing medical education practices in China and to present meaningful contrasts. Session duration was set at 45 min versus 90 min, with 45 min representing a standard session length in Chinese medical education and 90 min corresponding to a typical lecture or workshop block (34). Training frequency was defined as once a month versus once a week, reflecting differences in both session interval and overall course timeline, which in turn influences curriculum design and content organization (35). For qualitative attributes, levels were derived from existing practice patterns and expert consensus, ensuring each level represented a realistic and distinguishable training feature. The relevance and comprehensibility of all levels were confirmed during the qualitative interviews. This process yielded eight key attributes for use in the discrete choice experiment, as presented in Table 1.

**Table 1:** The description of attributes and levels.

Attribute	Definition	Level	Code
Instructor composition	Professionals who offer courses; Structure of the teaching staff	a. General practitioners	0
		b. General practitioner + clinical pharmacist	1
Teaching model	The model that the course is conducted	a. Lectures	0
		b. Lectures + case-based learning	1
Training location	Whether the training is organized online or on-site	a. On-site teaching	0
		b. Online teaching	1
Participant enrollment	Enrollment method of participants, i.e., the participants are involved individually or totally	a. Individual involvement	0
		b. Total involvement	1
Session duration	Time required for a session	a. 45 min	0
		b. 90 min	1
Training frequency	How often is each session conducted	a. Once a month	0
		b. Once a week	1
Assessment methods	The way to measure participant learning outcomes after training, including multiple-choice questions and case analysis	a. Multiple-choice questions	0
		b. Case analysis	1
Theoretical basis of the course	Main sources of pedagogical content in training courses and materials	a. Clinical practice guidelines	0
		b. Clinical medication brochure	1

All attributes in the DCE were categorical with two levels and were dummy-coded. For each attribute, the reference level was coded as 0 and the alternative level as 1, enabling straightforward interpretation of coefficients as the change in utility associated with moving from the reference to the alternative level.

#### Experiment design and choice set construction

Following the identification of relevant attributes and levels for the DCE, different attribute levels were systematically combined to generate hypothetical training implementation scenarios. If all possible combinations were included, a full factorial design with 8 attributes, each having 2 levels, would result in 2^8^ = 256 unique training alternatives. Pairing these alternatives into binary choice sets would yield a total of 32,640 possible combinations (i.e., 256 × 255/2), which would be impractical to present to respondents. To manage this complexity while preserving experimental efficiency, a fractional factorial design (FFD) was employed to construct a manageable set of choice scenarios. The design aimed to meet two essential criteria: (1) independence of attributes and (2) balanced representation, ensuring that each attribute level appeared with equal frequency across the experiment.

We used the orthogonal design function in IBM SPSS Statistics 25 to generate 12 distinct training implementation profiles that satisfied these criteria. A level-shifting approach was then applied to construct choice sets: for each profile, a second option was generated by incrementally shifting attribute levels (with wrap-around adjustment), creating paired alternatives. This process produced 12 binary choice sets. To minimize respondent fatigue and cognitive burden, the 12 choice sets were randomly divided into two versions of the questionnaire, with each version containing 6 DCE questions.

### Questionnaire

The questionnaire consisted of two main sections (see Supplementary File 1). The first section collected information on PCPs’ demographic characteristics and work-related information, including variables such as age, educational background, practice location, and clinic workload. The second section comprised six DCE questions. Each choice task presented respondents with two hypothetical training program alternatives, characterized by varying levels of key attributes, such as course format, training location, course duration, and training frequency.

To enhance respondents’ comprehension of the DCE, the questionnaire included detailed descriptions of each attribute and its corresponding levels. A sample DCE question was provided prior to the main choice tasks to familiarize participants with the format and structure of the experiment. This introductory example aimed to ensure clarity and reduce potential misunderstandings during the selection process. Table 2 presents the sample question included in the questionnaire.

**Table 2:** An example of discrete choice experiment task.

	Training plan A	Training plan B
Instructors composition	General practitioner	General practitioner + Clinical pharmacist
Teaching model	Lectures + Case-based learning	Lectures
Training location	Online teaching	On-site teaching
Participant enrollment	Individual involvement	Total involvement
Session duration	90 min	45 min
Training frequency	Once a week	Once a month
Assessment methods	multiple-choice questions	Case analysis
Theoretical basis of the course	Clinical practice guidelines	Clinical medication brochure
What is your choice?	☑	□

“√” indicates that the respondent selected Training plan A in the sample question. This choice reflects a preference for the specific combination of attribute levels presented in Option A after comparing both alternatives.

### Data collection

Data collection for this study was conducted over a three-month period, from October 10 to December 5, 2024, across multiple regions in eastern China, including Hangzhou, Cixi, Daishan, Suichang, and Kaihua. A mixed approach combining both convenience and purposive sampling techniques was employed to ensure broad and diverse participation while targeting specific demographic groups relevant to the study’s objectives.

The survey was administered using Questionnaire Star,^1^ an online survey platform operated by Changsha Ranxing Information Technology Co., Ltd. This platform was selected for its comprehensive functionality, including features for survey distribution, data collection, and analysis. The use of an online platform allowed for efficient outreach across a wide geographical area. Upon creation of the questionnaire, an online link was generated through Questionnaire Star, which facilitated the distribution process. The health administrators of each community health centers were contacted to help promote the survey and encourage participation.

To ensure the quality and reliability of the responses, several measures were incorporated into the survey’s design. First, a minimum time engagement requirement of 90 s was set before respondents could submit their responses to discourage rushed answers and ensure that participants consider each question carefully. Second, the survey was structured to require completion of all items before submission, and if any questions were left unanswered, an error message would prompt the respondent to fill in the required fields. Third, the survey included built-in response validation for key demographic questions, such as age and education level, to avoid inconsistencies or erroneous data entries. Fourth, to minimize the potential for careless or low-quality responses, screening questions were embedded within the survey to identify disengaged respondents. Moreover, the collected data were exported for cleaning and analysis, during which research assistants reviewed and flagged any anomalies or questionable responses for exclusion.

### Statistical analysis

The analytical procedure comprised two sequential phases. Demographic characterization was conducted using descriptive statistics via IBM SPSS Statistics 25.0. The DCE data underwent primary analysis through a mixed logit model implemented in Stata 17.0. The model was estimated using maximum simulated likelihood with 500 Halton draws. Consistent with random utility theory, the analytical framework presumes that respondents select alternatives offering maximum perceived utility. The utility function for each choice alternative is expressed as:


$$U_{\mathrm{ntj}}=V_{\mathrm{ntj}}+\varepsilon_{\mathrm{ntj}}$$

where *U_ntj_* denotes the total utility of alternative *j* for individual *n* in choice task *t*. This utility function incorporates two components: (1) *V_ntj_*, the systematic utility component determined by observable attributes, and (2) *ε_ntj_*, the stochastic error term capturing unobservable factors. The systematic utility component *V_ntj_* is specified as:


$$V_{\mathrm{ntj}}=\sum_{k}\beta_{k}X_{\mathrm{ktj}}$$

where *β_k_* represents the coefficients corresponding to attributes *X_ktj_*, which denote the attribute levels for alternative *j* in choice task *t*. Attribute levels underwent appropriate coding prior to analysis, with specific coding specifications detailed in Table 1. The coefficient *β_k_* provides information regarding both preference direction (through its sign) and preference intensity (through its magnitude) for the corresponding attribute level (25). Detailed specifications of the coding scheme, parameter distributions, and the exact model syntax are provided in Supplementary File 2.

To facilitate intuitive comprehension of attribute importance, Relative Importance Scores (RIS) were calculated. These scores derive from the maximum utility differential between attribute levels, weighted by their corresponding coefficients. Statistical significance of attribute coefficients (*p*-values) was systematically incorporated into RIS calculations to ensure valid interpretability of attribute importance hierarchies (24, 36).

Furthermore, scenario analysis was implemented to evaluate how attribute level modifications influence predicted choice probabilities. Predicted probabilities were calculated using the mean coefficient estimates from the mixed logit model, reflecting the preferences of an average respondent. We established a baseline scenario in which the weekly training option was assigned the low-preference level for all attributes, while the monthly option was assigned the low-preference level for all attributes except training frequency. For each attribute *k*, we then constructed an improved scenario by switching only that attribute in the weekly option to its high-preference level, leaving the monthly option unchanged. The utility for each option was calculated as described above. The probability of choosing the weekly option was then calculated using the binary logit formula:


$$P_{\text{weekly}}=\frac{e^{V_{\text{weekly}}}}{e^{V_{\text{weekly}}}+e^{V_{\text{monthly}}}}$$

## Results

### Participant characteristics

**Figure 1:**
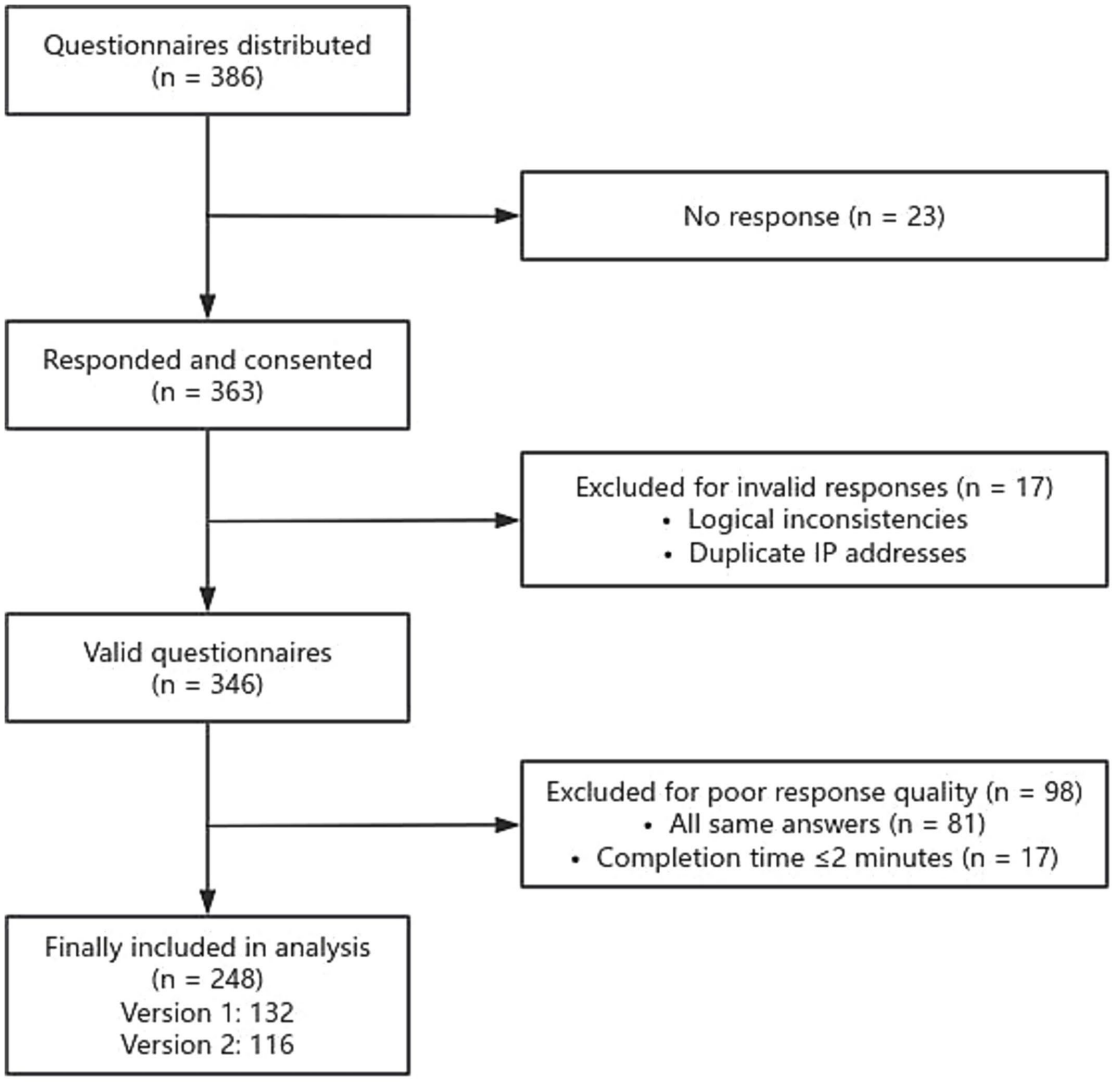
Questionnaire screening flow diagram.

The electronic questionnaires were distributed to 386 PCPs, and 363 PCPs responded and agreed to participate (response rate: 94.0%). After excluding 17 questionnaires with invalid responses, including logical inconsistencies in age and work experience and duplicate IP addresses, 346 valid questionnaires were retained. Of these 346 valid questionnaires, 81 were excluded due to providing uniform responses across all choice sets, indicating a lack of engagement or potential misunderstanding of the DCE tasks. An additional 17 respondents were excluded for completing the questionnaire in under 2 min, suggesting insufficient response quality. After applying these exclusion criteria, data from 248 PCPs (71.7%) were retained for analysis. The two versions of the questionnaire received responses from 132 and 116 participants, respectively. The detailed screening process is illustrated in Figure 1. No statistically significant differences were found in the demographic characteristics between the two groups (*p* > 0.05), indicating comparability across versions.

Participants ranged in age from 23 to 61 years, with a mean age of 37.7 years. The length of clinical practice varied from 1 to 42 years, with an average of 13.7 years. Most respondents specialized in general practice (61.3%), held the professional title of physician (46.4%), had attained a bachelor’s degree (81.5%), and were practicing in rural areas (65.3%). A majority had completed standardized residency training (63.7%) and were involved in family physician contracting services (69%). The average daily number of patients served by PCPs was 44. Detailed demographic characteristics of the participants are presented in Table 3. The demographic characteristics of our sample aligned broadly with those reported for primary care physicians in national health statistics, indicating an acceptable level of representativeness (37).

**Table 3:** Characteristics of respondents (*n* = 248).

Characteristics	Sub-groups	*N* (%)
Location	Suichang	65 (26.2)
	Daishan	60 (24.2)
	Cixi	60 (24.2)
	Kaihua	46 (18.5)
	Hangzhou	17 (6.9)
Gender	Male	125 (50.4)
	Female	123 (49.6)
Age, y	≤30	80 (32.3)
	31–40	71 (28.6)
	41–50	66 (26.6)
	>50	31 (12.5)
Duration of professional practice, y	≤10	113 (45.6)
	11–20	80 (32.3)
	21–30	36 (14.5)
	>30	19 (7.7)
Scope of practice	General practice	152 (61.3)
	Clinical specialty	64 (25.8)
	General practice plus clinical specialty	32 (12.9)
Professional title	Resident physician	115 (46.4)
	Attending physician	79 (31.9)
	Associate chief physician	38 (15.3)
	Chief physician	16 (6.5)
Educational background	High school or below	43 (17.3)
	Bachelor’s degree	202 (81.5)
	Master’s degree or above	3 (1.2)
Participation in standardized residency training	Yes	158 (63.7)
	No	90 (36.3)
Involvement in family doctor contract services	Yes	171 (69)
	No	77 (31)
Geographical location of the institution	Rural	162 (65.3)
	Urban	86 (34.7)
Nature of the employing institution	General hospitals	9 (3.6)
	Township health center	110 (44.4)
	Community health service center	62 (25)
	Village clinic	67 (27)
Average daily patient encounters (patients/day)	≤50	181 (73)
	51–100	55 (22.2)
	>100	12 (4.8)

### Preferences of PCPs for training implementation

The preference model was based on a total of 2,976 choice observations collected from 248 respondents. Sensitivity analyses compared the main analytic sample (*n* = 248, after excluding low-quality responses) with the full sample (*n* = 346). The direction of preferences for all attributes remained consistent between the two models. However, coefficients for significant attributes were substantially larger in the main analysis (e.g., training frequency: *β* = −0.86 vs. −0.35; session duration: *β* = −0.62 vs. −0.33), and model fit improved (McFadden’s pseudo-*R*^2^ increased from 0.046 to 0.132). Moreover, the preference for multidisciplinary instructors became statistically significant only after exclusion, while assessment method lost significance. These findings indicate that low-quality responses introduce noise and attenuate true preference estimates, supporting the validity of our exclusion criteria in reducing bias (see Supplementary Table 1 for full results).

Results from the mixed logit model indicated that five out of eight attributes demonstrated statistically significant effects relative to their respective reference levels, as detailed in Table 4. Based on the magnitude of their statistical significance, the attributes were ranked in the following order of importance: training frequency (37.29%), session duration (27.23%), instructor composition (16.29%), participant enrollment (10.23%), and theoretical basis of the course (8.95%). Besides, the teaching model, location, and assessment methods did not exhibit statistically significant effects on preferences (*p* > 0.05).

**Table 4:** Mixed logit model estimating PCPs’ preferences for attributes.

Attributes and levels	*β*	95%CI		SD	95%CI	
		Lower	Upper		Lower	Upper
Instructors composition (Ref: General practitioner)						
General practitioner + clinical pharmacist	0.373*	0.112	0.635	0.776*	0.396	1.156
Teaching model (Ref: lectures)						
Lectures + Case-based learning	−0.008	−0.226	0.210	−0.068	−0.559	0.424
Training location (Ref: on-site teaching)						
Online teaching	0.013	−0.247	0.273	1.525*	1.142	1.908
Participant enrollment (Ref: individual involvement)						
Total involvement	−0.235*	−0.470	0.000	−0.734*	−1.082	−0.387
Session duration (Ref: 45 min)						
90 min	−0.624*	−0.875	−0.374	0.857*	0.526	1.188
Training frequency (Ref: once a month)						
Once a week	−0.855*	−1.121	−0.589	1.169*	0.820	1.518
Assessment methods (Ref: multi-choice questions)						
Case analysis	0.093	−0.092	0.278	0.330	−0.160	0.820
Theoretical basis of the course (Ref: clinical practice guideline)						
Clinical medication brochure	−0.205*	−0.390	−0.020	−0.302	−0.767	0.162
Log-likelihood	−894.515	Pseudo-R^2^			0.132	
AIC	1,823.030	RLH mean			0.526	
BIC	1,925.002					

Levels that were statistically significant at 5% level were shown by “^*^” and displayed in bold.

Ref, reference level; SD, standard deviation; 95% CI, confidence interval.

Among all factors, the training frequency emerged as the most influential determinant, with a relative importance score of 37.29%. Compared to a monthly training schedule, a weekly frequency significantly reduced preference among PCPs, nearly doubling the decline in utility (*β* = −0.86; 95% CI: −1.12 to −0.59). The second most influential factor was training duration, with a relative importance score of 27.23%; shorter sessions were clearly preferred over longer ones (*β* = −0.62; 95% CI: −0.87 to −0.37). Additionally, a collaborative instructional model involving both general practitioners and clinical pharmacists was associated with a significantly higher preference (*β* = 0.37; 95% CI: 0.11–0.64). By comparison, the attributes related to participant enrollment (*β* = −0.24; 95% CI: −0.47 to 0.00) and theoretical basis (*β* = −0.21; 95% CI: −0.39 to −0.02) were perceived as relatively less important by respondents.

The mixed logit model demonstrated good fit (McFadden’s pseudo-*R*^2^ = 0.132) and internal validity (mean RLH = 0.526). Robustness checks using alternative model specifications confirmed that the relative importance rankings of the five key attributes remained consistent with the main analysis (Supplementary File 3).

**Figure 2:**
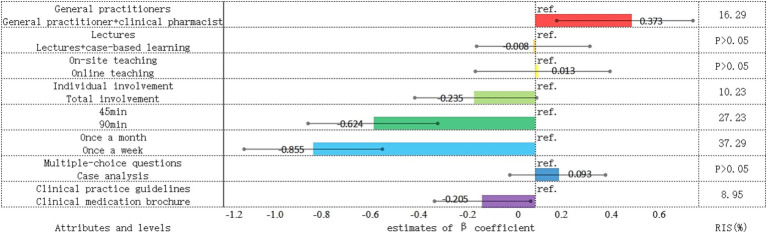
PCPs’ preferences for implementing training programs. *β*, the average preferences of the study population; Ref, reference level; RIS, relative importance scores.

Random parameters were incorporated into the mixed logit model to estimate standard deviations (SDs), allowing for the assessment of preference heterogeneity among respondents. The results indicated that only five attributes (i.e., instructor composition, training location, target population, duration of training sessions, and training frequency) exhibited statistically significant SD estimates. This suggests that substantial heterogeneity existed in respondents’ preferences regarding these aspects of training program. The estimated coefficients and the relative importance scores for each attribute, derived from the mixed logit model, are presented in Figure 2.

### Changes in selection rates for training implementation

**Figure 3:**
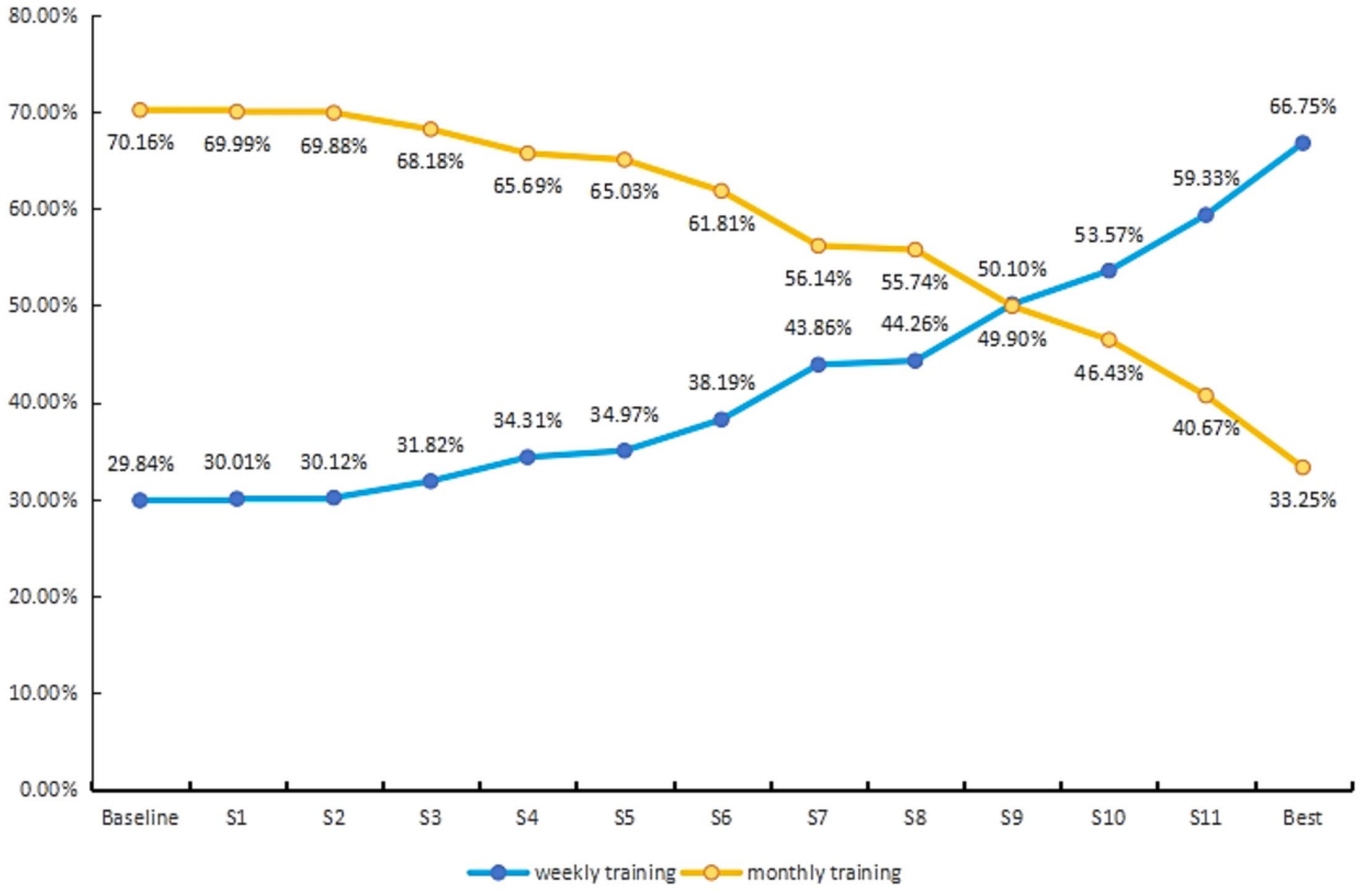
Changes in selection rates for training implementation. The baseline scenario is characterized by low-preference levels (general practitioners, lectures + case-based learning, onsite teaching, total involvement, 90 min, multiple-choice questions, and clinical medication brochure); S1 = Lectures; S2 = Online teaching; S3 = Case analysis; S4 = Clinical practice guidelines; S5 = Individual involvement; S6 = General practitioners + clinical pharmacists; S7 = ‘General practitioners + clinical pharmacists’ + Individual involvement; S8 = 45 min; S9 = Individual involvement + 45 min; S10 = ‘General practitioners + clinical pharmacists’ + 45 min; S11 = ‘General practitioners + clinical pharmacists’ + Individual involvement + 45 min; Best = ‘General practitioners + clinical pharmacists’ + lectures + online teaching + individual involvement + 45 min + case analysis + clinical practice guidelines.

Based on the mixed logit model estimates, Figure 3 illustrates the dynamic changes in choice probabilities across different training implementation scenarios. In the baseline scenario, which consisted of the least preferred attribute levels (instruction by general practitioners alone, lectures combined with case-based learning, onsite delivery, mandatory group participation, 90-min sessions, assessment via multiple-choice questions, and reliance on the clinical medication brochure), the predicted choice probabilities were 70.16% for monthly training and 29.84% for weekly training, reflecting primary care physicians’ inherent aversion to high-frequency training formats.

By adjusting each attribute of the weekly training option individually toward the more preferred levels, we quantified the marginal contribution of each feature to increasing participation willingness. The results showed that, among all single-attribute optimizations, reducing session duration from 90 to 45 min yielded the greatest marginal benefit, increasing the choice probability of weekly training by 14.42 percentage points to 44.26%. This finding indicates that when resources are constrained and simultaneous optimization of multiple design elements is infeasible, prioritizing the compression of single-session duration is the most efficient strategy for enhancing physicians’ willingness to participate.

Notably, the effect of combined improvements substantially exceeded that of single-attribute adjustments. When both shorter session duration and individual voluntary enrollment were implemented together, the choice probability of weekly training approached that of the monthly option. Further optimization encompassing instructor composition, teaching format, assessment methods, and theoretical foundation elevated the predicted choice probability of weekly training to 66.75%, more than double the baseline rate. These results suggest that although time burden is a core barrier to participation, systematic multidimensional optimization can effectively mitigate physicians’ initial resistance to high-frequency training. Figure 3 presents the cumulative effects of various attribute combinations, offering actionable insights for phased and targeted enhancement of training program design.

## Discussion

This study utilized a DCE to investigate PCPs’ preferences regarding training programs, with a particular focus on prescribing and deprescribing in the context of multimorbidity. Distinct from prior research, which often centers on general training needs or outcomes, this study emphasizes the structured design of implementation components by standardizing training content. This approach enables a more precise identification of the optimal configuration of training programs. Moreover, the study extends the application of DCE methodology to the field of medical education, an area where such approaches remain underutilized. The results underscore the multifaceted nature of PCPs’ preferences, which span key attributes such as instructor composition, participant enrollment, training duration and frequency, and the theoretical basis of the course.

When implementing training programs, PCP demonstrated a clear preference for sessions facilitated by a multidisciplinary team comprising general practitioners and clinical pharmacists, conducted individually rather than in groups, held on a monthly basis, limited to 45 min in duration, and grounded in clinical practice guidelines. Among the various training attributes evaluated, session frequency and duration emerged as the most influential factors shaping PCPs’ preferences. Notably, PCPs expressed a preference for less frequent and shorter training sessions which contrasts with initial expectations. The observed preference for less frequent and shorter training sessions aligns with the demanding clinical workloads faced by PCPs (38). Subgroup analyses stratified by the sample medians provided empirical support for this interpretation. Physicians with higher workload exhibited substantially stronger aversion to weekly training and 90-min sessions (see Supplementary Tables 5, 6). These findings indicate that time pressure intensifies the need for time-efficient formats (30, 39). Although more experienced physicians also showed stronger preference for monthly over weekly training, the difference between workload groups was more pronounced, and no consistent pattern emerged for session duration by experience. This suggests that current workload may be a more salient determinant of training format preferences than cumulative professional experience. However, this reluctance may be mitigated through targeted adjustments in training design. For instance, optimizing the composition of the instructional team, streamlining enrolment procedures, enhancing the relevance and practicality of training materials, and maintaining concise session durations could enhance the acceptability and feasibility of more frequent training sessions among PCPs.

The mode of involvement in training programs plays a critical role in influencing PCPs’ willingness to participate. As physicians are increasingly expected to attend multiple training initiatives, the cumulative time commitment can lead to training fatigue and reduced enthusiasm for participation (40). Moreover, the growing accessibility of medical knowledge through digital platforms has fostered a stronger preference for self-directed learning among PCPs (31), as it offers greater flexibility and autonomy in managing their professional development. This may account for the observed preference for individual participation over mandatory group-based or all-staff training formats, which are perceived as less adaptable to individual schedules and learning needs.

With regard to the composition of instructional staff, PCPs expressed a preference for training sessions delivered jointly by general practitioners and clinical pharmacists, a finding consistent with our initial expectations. Given the specialized nature of prescribing and deprescribing, the inclusion of pharmacists as co-instructors is perceived as particularly valuable, offering targeted expertise and clinical depth. This interdisciplinary approach not only enhances the comprehensiveness and quality of the training but also ensures that participants receive guidance grounded in real-world pharmacological practice (41). Moreover, exposure to role models from relevant professional backgrounds, particularly those who are both knowledgeable and engaging, has been shown to significantly enrich the educational experience. Such exposure supports the acquisition of advanced prescribing competencies and increases participant engagement and interest in the training content (42). Evidence also indicates that collaborative practice between pharmacists and physicians improves prescribing accuracy and promotes safer prescribing behaviors within the healthcare system (43, 44).

While the average preference for training location among respondents did not reach statistical significance, notable heterogeneity was observed, suggesting that only a subset of PCPs expressed strong preferences regarding training venue. Previous studies have reported comparable levels of participant engagement and knowledge retention between on-site and online training modalities (29, 45). Although online training offers clear advantages in terms of convenience and scheduling flexibility, on-site formats continue to provide unique benefits, particularly in promoting interpersonal interaction, collaboration among trainees, and real-time engagement with instructors (32, 46). Furthermore, disparities in digital literacy and comfort with technology, especially among older PCPs, can limit the accessibility and effectiveness of online training. As a result, despite growing acceptance of online learning, it is unlikely to serve as a full substitute for in-person training in the near term (14). Nonetheless, online formats can play a valuable supplementary role when their strengths are strategically leveraged—for instance, by providing recorded lectures, digital course materials, or follow-up modules that enhance and reinforce on-site learning experiences (45, 46).

Given the demanding clinical workloads and familial obligations commonly faced by PCPs, training programs must prioritize feasibility and voluntary participation. Our findings suggest that optional, self-directed enrollment may be more acceptable than compulsory formats, promoting greater engagement and sustained commitment. Moreover, the inclusion of a multidisciplinary teaching faculty, particularly the active involvement of clinical pharmacists, can enhance the depth and credibility of training content. This interdisciplinary approach is critical to ensuring both pedagogical rigor and practical relevance in clinical practice. To maximize engagement and accessibility, curriculum design should emphasize flexible scheduling. A hybrid model combining online and offline modalities appears particularly effective. Live sessions facilitate real-time interaction, while digital platforms provide access to recorded lectures and learning materials. This format accommodates PCPs’ time constraints, supports self-paced learning, expands geographical reach, reduces logistical barriers, and ultimately enhances participation and knowledge uptake.

Several key considerations are essential for the successful implementation of such training programs. First, modular and flexible learning formats should be prioritized. Structuring the curriculum into smaller, focused “mini-training” modules enables physicians to incorporate learning into their routine clinical practice, thereby mitigating time and regional limitations. These micro-courses, delivered via a blended learning platform, offer a pragmatic solution for continuous professional development. Second, the establishment of a multidisciplinary and diverse teaching team is critical. Involving experts from various fields (such as clinical pharmacology, geriatrics, and pharmacy) enriches the learning experience by providing multiple clinical perspectives, thereby enhancing both the academic rigor and real-world applicability of the content. Third, the adoption of demand-driven strategies and incentive mechanisms can significantly increase program uptake. Providing formal certification or continuing education credits serves as a motivational tool. Furthermore, ongoing feedback from participants and regular assessments of clinical demand can inform curriculum refinement and ensure sustained relevance. Lastly, robust assessment strategies are necessary to evaluate the effectiveness of the training. Periodic evaluations, such as quizzes, case-based discussions, simulated prescriptions, or prescription audits, can help assess learner competence, reinforce knowledge retention, and inform continuous curriculum improvement.

This study has several limitations. First, the geographic focus on eastern China may constrain the generalizability of the findings, and replication in other regions is needed to validate the results. Second, the DCE design did not include an opt-out option and implicitly assumed respondents’ willingness to participate in training, limiting the ability to assess actual training intent or identify barriers to engagement. Third, due to space limitations in the survey instrument, certain potentially relevant attributes and levels were excluded, which may have resulted in the omission of key factors influencing PCP preferences. Fourth, this study captures stated preferences rather than revealed preferences, and a gap may exist between hypothetical choices and actual behavior. Therefore, our findings provide theoretical guidance for training design but require future validation against real-world participation and prescribing outcomes. Future research should consider a more comprehensive range of implementation attributes and adopt longitudinal designs to assess the long-term impact of training on prescribing and deprescribing behaviors and patient outcomes.

## Conclusion

This study elucidates PCPs’ preferences for training programs focused on prescribing and deprescribing in the context of multimorbidity. The results indicate that PCPs prefer training programs characterized by low frequency, short duration, individual involvement, instruction by a multidisciplinary team, and content grounded in clinical practice guidelines. These insights offer a theoretical foundation for the future design of training interventions, with the potential to enhance their feasibility, acceptability, and participation among PCPs.

## Data Availability

The datasets presented in this study can be found in online repositories. The names of the repository/repositories and accession number(s) can be found in the article/Supplementary material.

## Footnotes

1
https://www.wjx.cn/

## References

[1] Tian F Chen Z Wu J. Prevalence of polypharmacy and potentially inappropriate medications use in elderly Chinese patients: a systematic review and meta-analysis. Front Pharmacol. (2022) 13:862561. doi: 10.3389/fphar.2022.862561 35795561 PMC9251439

[2] Bodenheimer T Wagner EH Grumbach K. Improving primary care for patients with chronic illness. JAMA. (2002) 288:1775–9. doi: 10.1001/jama.288.14.1775 12365965

[3] Zhou X Han L Farmer A Yao M Xia Y Yan M . Challenges and barriers to physician decision-making for prescribing and deprescribing among patients with multimorbidity in eastern China’s primary care settings: a qualitative study. BMJ Open. (2025) 15:e095063. doi: 10.1136/bmjopen-2024-095063 PMC11808900 39922587

[4] Marengoni A Angleman S Melis R Mangialasche F Karp A Garmen A . Aging with multimorbidity: a systematic review of the literature. Ageing Res Rev. (2011) 10:430–9. doi: 10.1016/j.arr.2011.03.003 21402176

[5] Nie XY Dong XX Lu H Li DL Zhao CH Huang Y . Multimorbidity patterns and the risk of falls among older adults: a community-based study in China. BMC Geriatr. (2024) 24:660. doi: 10.1186/s12877-024-05245-1 39112944 PMC11304791

[6] Skou ST Mair FS Fortin M Guthrie B Nunes BP Miranda JJ . Multimorbidity. Nat Rev Dis Primers. (2022) 8:48. doi: 10.1038/s41572-022-00376-4 35835758 PMC7613517

[7] National Health Commission of the People’s Republic of China. Statistical bulletin on the development of health care in China (2023). Available online at: https://www.nhc.gov.cn/guihuaxxs/c100133/202408/0c53d04ede9e4079afff912d71b5131c.shtml (Accessed May 10, 2025).

[8] Pazan F Wehling M. Polypharmacy in older adults: a narrative review of definitions, epidemiology and consequences. Eur Geriatr Med. (2021) 12:443–52. doi: 10.1007/s41999-021-00479-3 33694123 PMC8149355

[9] Endocrinology and Metabolism Branch of Chinese Association of Geriatric Research & Committee of Clinical Toxicology of Chinese Society of Toxicology. Expert consensus on the secure management of polypharmacy in the elderly. Chin Gen Pract. (2018) 21:3533–44. doi: 10.12114/j.issn.1007-9572.2018.00.225

[10] Dovjak P. Polypharmacy in elderly people. Wien Med Wochenschr. (2022) 172:109–13. doi: 10.1007/s10354-021-00903-0 35006518

[11] National Health Commission of the People’s Republic of China. Opinions on strengthening the pharmaceutical management of medical institutions and promoting the rational use of medicines. Available online at: https://www.nhc.gov.cn/yzygj/c100068/202002/fdb7240014db4d49827ca25bbb41b177.shtml (Accessed May 10, 2025).

[12] Lavan AH Gallagher PF O'Mahony D. Methods to reduce prescribing errors in elderly patients with multimorbidity. Clin Interv Aging. (2016) 11:857–66. doi: 10.2147/CIA.S80280 27382268 PMC4922820

[13] Celebi N Weyrich P Riessen R Kirchhoff K Lammerding-Köppel M. Problem-based training for medical students reduces common prescription errors: a randomised controlled trial. Med Educ. (2009) 43:1010–8. doi: 10.1111/j.1365-2923.2009.03452.x 19769651

[14] Yo EC Witjaksono AN Fitriani DY Werdhani RA Parikesit D. Evaluating knowledge retention and perceived benefits of medical webinar for professional development among Indonesian physicians. Korean J Med Educ. (2021) 33:381–91. doi: 10.3946/kjme.2021.206 34875154 PMC8655361

[15] McEvoy MD Fowler LC Robertson A Gelfand BJ Fleming GM Miller B . Comparison of two learning modalities on continuing medical education consumption and knowledge acquisition: a pilot randomized controlled trial. J Educ Perioper Med. (2021) 23:E668. doi: 10.46374/volxxiii_issue3_mcevoy 34631966 PMC8491639

[16] Esparbes L Escourrou E Birebent J Buscail L Dupouy J Durliat S . Development and validation of a training course on proton pump inhibitor deprescription for general practitioners in a rural continuing medical education program: a pilot study. BMC Med Educ. (2024) 24:1221. doi: 10.1186/s12909-024-06215-2 39465370 PMC11514963

[17] Moore TR Chusan YAC Pachucki M Kim B. A participatory systems approach for visualizing and testing implementation strategies and mechanisms: evidence adoption in community coalitions. Implement Sci Commun. (2025) 6:96. doi: 10.1186/s43058-025-00788-9 41035014 PMC12487054

[18] Eccles MP Mittman BS. Welcome to implementation science. Implementation Sci. (2006) 1:1. doi: 10.1186/1748-5908-1-1

[19] Curran GM Bauer M Mittman B Pyne JM Stetler C. Effectiveness-implementation hybrid designs: combining elements of clinical effectiveness and implementation research to enhance public health impact. Med Care. (2012) 50:217–26. doi: 10.1097/MLR.0b013e3182408812 22310560 PMC3731143

[20] Leeman J Birken SA Powell BJ Rohweder C Shea CM. Beyond "implementation strategies": classifying the full range of strategies used in implementation science and practice. Implement Sci. (2017) 12:125. doi: 10.1186/s13012-017-0657-x 29100551 PMC5670723

[21] Santos Martiniano C de Castro ME Barros de Souza M Alves Coelho A Arcêncio RA Fronteira I . The gap between training and practice of prescribing of drugs by nurses in the primary health care: a case study in Brazil. Nurse Educ Today. (2016) 36:304–9. doi: 10.1016/j.nedt.2015.07.017 26277426

[22] Cleland J Porteous T Skåtun D. What can discrete choice experiments do for you? Med Educ. (2018) 52:1113–24. doi: 10.1111/medu.13657 30259546

[23] Heidenreich S Finney Rutten LJ Miller-Wilson LA Jimenez-Moreno C Chua GN Fisher DA. Colorectal cancer screening preferences among physicians and individuals at average risk: a discrete choice experiment. Cancer Med. (2022) 11:3156–67. doi: 10.1002/cam4.4678 35315224 PMC9385595

[24] Yang J Ma B Chen S Huang Y Wang Y Chen Y . Nurses’ preferences for working in Uber-style ‘internet plus’ nursing services: a discrete choice experiment. Int J Nurs Stud. (2025) 161:104920. doi: 10.1016/j.ijnurstu.2024.104920 39378739

[25] Tian Z Guo W Zhai M Li H. Job preference of preventive medicine students during the COVID-19 pandemic: a discrete choice experiment survey in Shandong Province, China. BMC Med Educ. (2023) 23:890. doi: 10.1186/s12909-023-04873-2 38012762 PMC10680353

[26] Phillips KA Johnson FR Maddala T. Measuring what people value: a comparison of “attitude” and “preference” surveys. Health Serv Res. (2002) 37:1659–79. doi: 10.1111/1475-6773.01116 12546291 PMC1464045

[27] Xie Z Liu H Or C. A discrete choice experiment to examine the factors influencing consumers’ willingness to purchase health apps. Mhealth. (2023) 9:21. doi: 10.21037/mhealth-22-39 37492118 PMC10364011

[28] Zhou L Wei X Wu Y Deng X Xu M Shang X . Preferences for training needs of village doctors in China: a systematic review. Fam Pract. (2024) 41:874–82. doi: 10.1093/fampra/cmad063 37300310

[29] Thepwongsa I Kirby CN Schattner P Piterman L. Online continuing medical education (CME) for GPs: does it work? A systematic review. Aust Fam Physician. (2014) 43:717–21. 25286431

[30] Cai S Jiang X Hua Y Qian D Wang X Pan T. Discrete choice experiment on the preferences for continuing medical education training programs among primary health care physicians in China. BMC Med Educ. (2025) 25:315. doi: 10.1186/s12909-025-06828-1 40011922 PMC11866800

[31] Devine OP Harborne AC Horsfall HL Joseph T Marshall-Andon T Samuels R . The analysis of teaching of medical schools (AToMS) survey: an analysis of 47,258 timetabled teaching events in 25 UK medical schools relating to timing, duration, teaching formats, teaching content, and problem-based learning. BMC Med. (2020) 18:126. doi: 10.1186/s12916-020-01571-4 32404194 PMC7222546

[32] Mueller MR Croghan IT Schroeder DR . Physician preferences for online and in-person continuing medical education: a cross-sectional study. BMC Med Educ. (2024) 24:1142. doi: 10.1186/s12909-024-06046-1 39402550 PMC11476101

[33] Proctor EK Powell BJ McMillen JC. Implementation strategies: recommendations for specifying and reporting. Implement Sci. (2013) 8:139. doi: 10.1186/1748-5908-8-139 24289295 PMC3882890

[34] Klement A Ibs T Longard S Klinkhart C Frese T Heise M. “Corona-debriefing”: concept and pilot testing of a 90-minute workshop for undergraduate-education and specialist-training in family medicine. GMS. J Med Educ. (2020) 37:Doc95. doi: 10.3205/zma001388 PMC7740013 33364374

[35] Khalafi A Fallah Z Sharif-Nia H. The effect of spaced learning on the learning outcome and retention of nurse anesthesia students: a randomized-controlled study. BMC Med Educ. (2024) 24:322. doi: 10.1186/s12909-024-05290-9 38515084 PMC10958887

[36] Mansfield C Gebben DJ Sutphin J Tepper SJ Schwedt TJ Sapra S . Patient preferences for preventive migraine treatments: a discrete-choice experiment. Headache. (2019) 59:715–26. doi: 10.1111/head.13498 30861110

[37] National Health Commission of China. 2024 China Health Statistics Yearbook. Beijing: Peking Union Medical College Press (2025).

[38] Schäfer WLA van den Berg MJ Groenewegen PP. The association between the workload of general practitioners and patient experiences with care: results of a cross-sectional study in 33 countries. Hum Resour Health. (2020) 18:76. doi: 10.1186/s12960-020-00520-9 33066776 PMC7565810

[39] Reis T Faria I Serra H Xavier M. Barriers and facilitators to implementing a continuing medical education intervention in a primary health care setting. BMC Health Serv Res. (2022) 22:638. doi: 10.1186/s12913-022-08019-w 35562695 PMC9099036

[40] Brass LF Akabas MH. The national MD-PhD program outcomes study: relationships between medical specialty, training duration, research effort, and career paths. JCI Insight. (2019) 4:e133009. doi: 10.1172/jci.insight.133009 31578310 PMC6795497

[41] Lam TP Lam YY. Medical education reform: the Asian experience. Acad Med. (2009) 84:1313–7. doi: 10.1097/ACM.0b013e3181b18189 19707080

[42] Atmann O Torge M Schneider A. The “General practitioner learning stations”-development, implementation and optimization of an innovative format for sustainable teaching in general practice. BMC Med Educ. (2021) 21:622. doi: 10.1186/s12909-021-03057-0 34915875 PMC8680029

[43] Noble C Brazil V Teasdale T Forbes M Billett S. Developing junior doctors’ prescribing practices through collaborative practice: sustaining and transforming the practice of communities. J Interprof Care. (2017) 31:263–72. doi: 10.1080/13561820.2016.1254164 28140691

[44] Barbosa Detoni K Lopes André A Rezende CP Furtado BT de Araújo Medina Mendonça S Ramalho-de-Oliveira D. Interprofessional education for shared decision making in drug therapy: a scoping review. J Interprof Care. (2023) 37:491–503. doi: 10.1080/13561820.2022.2039598 35285394

[45] O’Brien Pott M Blanshan AS Huneke KM Baasch Thomas BL Cook DA. What influences choice of continuing medical education modalities and providers? A National Survey of U.S. physicians, nurse practitioners, and physician assistants. Acad Med. (2021) 96:93–100. doi: 10.1097/ACM.0000000000003758 32969838

[46] Delungahawatta T Dunne SS Hyde S Halpenny L McGrath D O’Regan A . Advances in e-learning in undergraduate clinical medicine: a systematic review. BMC Med Educ. (2022) 22:711. doi: 10.1186/s12909-022-03773-1 36207721 PMC9540295

