# Supplementary material for: Preferences for implementing training program among primary care physicians in prescribing and deprescribing for patients with multimorbidity: a discrete choice experiment
Source: Front Med (Lausanne). 2026 Mar 13;13:1795722. doi: 10.3389/fmed.2026.1795722 (PMC13021398; doi:10.3389/fmed.2026.1795722)
Supplement: Supplementary file 1 [file Supplementary_file_1.docx]

**Supplementary file**

**Prescribing and Deprescribing Training Preferences for Patients with Multimorbidity Among Primary Care Physicians: a survey**

Dear physician,

Greetings! We are members of the Prescribing and deprescribing for Multimorbidity Training research team at Zhejiang University. We are conducting a survey regarding primary care physicians’ preferences for participating in prescribing and deprescribing training for patients with multimorbidity. The purpose of this survey is to understand your preferences for implementing such training. By collecting your valuable insights, we aim to provide an important reference basis for developing future implementation strategies, thereby enhancing the quality of medication therapy for multimorbidity in primary care settings.

Therefore, we sincerely invite you to participate in this survey. The questionnaire is anonymous, and there are no right or wrong answers. Please fill it out based on your actual practices and perspectives, selecting the most appropriate options. Completing this survey will take approximately 3-4 minutes. We assure you of the confidentiality of your information; all data will be kept secure and not disclosed. Please note that participation in this survey is entirely voluntary; you are also free to decide whether to participate and may withdraw at any time. We sincerely appreciate your support and participation!

**Informed consent for participation in this survey**

□ I have read the above information and voluntarily agree to participate in the survey.
□ I decline to participate in this survey.

**Part I: Basic information**

1. Your gender:

□Male □Female

1. Your age：________（y）
2. Duration of professional practice：________（y）
3. Your scope of practice:

□General practice

□General practice plus clinical specialty

□Clinical specialty

1. Your professional title:

□Attending physician

□Resident physician

□Associate chief physician

□Chief physician

1. Your educational background:

□High school or below

□Bachelor’s degree

□Master’s degree or above

1. Have you ever participated in standardized residency training:

□Yes □No

1. Are you involved in family doctor contract services:

□Yes □No

1. Geographical location of your institution:

□Rural □Urban

1. Nature of your employing institution:

□Community health service center

□Township health center

□Village clinic

□General hospital

1. Your average daily patient encounters in the past month：_______（patients/day）

**Part Ⅱ：Primary Care Physicians’ Preferences for Training Programs in Prescribing and Deprescribing for Multimorbidity**

This section aims to understand your preferences regarding prescribing and deprescribing training, which will inform the development and optimization of medication regimens for patients with multimorbidity. Your input is crucial for enhancing primary care quality and improving treatment outcomes in this patient population. Completion is estimated to take 2-3 minutes. All responses will be maintained under strict confidentiality.

1. **The description of attributes and levels**

| **Attribute** | **Definition** | **Level** |
| --- | --- | --- |
| **Instructor composition** | Professionals who offer courses;  Structure of the teaching staff | 1. General practitioners 2. General practitioner   +clinical pharmacist |
| Teaching model | The model that the course is conducted | a)Lectures  b)Lectures+case-based  learning |
| Training location | Whether the training is organized online or on-site | 1. On-site teaching 2. Online teaching |
| Participant enrollment | Enrollment method of participants, i.e., the participants are involved individually or totally | 1. Individual involvement 2. Total involvement |
| Session duration | Time required for a session | 1. 45min 2. 90min |
| Training frequency | How often is each session conducted | 1. Once a month 2. Once a week |
| Assessment methods | The way to measure participant learning outcomes after training, including multiple-choice questions and case analysis | 1. Multiple-choice questions 2. Case analysis |
| Theoretical basis of the course | Main sources of pedagogical content in training courses and materials | 1. Clinical practice guidelines 2. Clinical medication brochure |

**（2）Scenario-Based Simulation:**

The following section presents 6 choice questions. Each question shares an identical scenario: Suppose you were to receive training on prescribing and deprescribing for patients with multimorbidity. You may choose between two instructional formats that yield identical clinical outcomes but differ in their educational processes. We ask you to consider which format you would prefer.

**The sample question item:**

|  | Training plan A | Training plan B |
| --- | --- | --- |
| Instructors composition | General practitioner | General practitioner  +Clinical pharmacist |
| Teaching model | Lectures+Case-based learning | Lectures |
| Training location | Online teaching | On-site teaching |
| Participant enrollment | Individual involvement | Total involvement |
| Session duration | 90 min | 45 min |
| Training frequency | Once a week | Once a month |
| Assessment methods | multiple-choice questions | Case analysis |
| Theoretical basis of the course | Clinical practice guidelines | Clinical medication brochure |
| **What is your choice?** | ☑ | □ |

“√” indicates that the respondent selected Training plan A in the sample question. This choice reflects a preference for the specific combination of attribute levels presented in Option A after comparing both alternatives.

In the following six hypothetical scenarios, Training Plan A and Training Plan B are comparable but differ in content. After careful comparison, **please select your preferred plan by marking “✓” in the “□”. Note: There are no correct or incorrect choices**—only differences in training processes, required effort, and time commitment. Please indicate which plan you would prefer.

| **S1** | **Training plan A** | **Training plan B** |
| --- | --- | --- |
| Instructors composition | General practitioners | General practitioner  +clinical pharmacist |
| Teaching model | Lectures+case-based learning | Lectures |
| Training location | Online teaching | On-site teaching |
| Participant enrollment | Individual involvement | Total involvement |
| Session duration | 90min | 45min |
| Training frequency | Once a week | Once a month |
| Assessment methods | Case analysis | Multiple-choice questions |
| Theoretical basis of the course | Clinical practice guidelines | Clinical medication brochure |
| **What is your choice?** | □ | □ |

| **S2** | **Training plan A** | **Training plan B** |
| --- | --- | --- |
| Instructors composition | General practitioner  +clinical pharmacist | General practitioners |
| Teaching model | Lectures | Lectures+case-based learning |
| Training location | Online teaching | On-site teaching |
| Participant enrollment | Individual involvement | Total involvement |
| Session duration | 90min | 45min |
| Training frequency | Once a week | Once a month |
| Assessment methods | Multiple-choice questions | Case analysis |
| Theoretical basis of the course | Clinical medication brochure | Clinical practice guidelines |
| **What is your choice?** | □ | □ |

| **S3** | **Training plan A** | **Training plan B** |
| --- | --- | --- |
| Instructors composition | General practitioner  +clinical pharmacist | General practitioners |
| Teaching model | Lectures | Lectures+case-based learning |
| Training location | Online teaching | On-site teaching |
| Participant enrollment | Total involvement | Individual involvement |
| Session duration | 45min | 90min |
| Training frequency | Once a month | Once a week |
| Assessment methods | Case analysis | Multiple-choice questions |
| Theoretical basis of the course | Clinical medication brochure | Clinical practice guidelines |
| **What is your choice?** | □ | □ |

| **S4** | **Training plan A** | **Training plan B** |
| --- | --- | --- |
| Instructors composition | General practitioners | General practitioner  +clinical pharmacist |
| Teaching model | Lectures | Lectures+case-based learning |
| Training location | On-site teaching | Online teaching |
| Participant enrollment | Individual involvement | Total involvement |
| Session duration | 90min | 45min |
| Training frequency | Once a month | Once a week |
| Assessment methods | Case analysis | Multiple-choice questions |
| Theoretical basis of the course | Clinical medication brochure | Clinical practice guidelines |
| **What is your choice?** | □ | □ |

| **S5** | **Training plan A** | **Training plan B** |
| --- | --- | --- |
| Instructors composition | General practitioners | General practitioner  +clinical pharmacist |
| Teaching model | Lectures+case-based learning | Lectures |
| Training location | On-site teaching | Online teaching |
| Participant enrollment | Total involvement | Individual involvement |
| Session duration | 45min | 90min |
| Training frequency | Once a week | Once a month |
| Assessment methods | Case analysis | Multiple-choice questions |
| Theoretical basis of the course | Clinical medication brochure | Clinical practice guidelines |
| **What is your choice?** | □ | □ |

| **S6** | **Training plan A** | **Training plan B** |
| --- | --- | --- |
| Instructors composition | General practitioners | General practitioner  +clinical pharmacist |
| Teaching model | Lectures | Lectures+case-based learning |
| Training location | On-site teaching | Online teaching |
| Participant enrollment | Individual involvement | Total involvement |
| Session duration | 45min | 90min |
| Training frequency | Once a month | Once a week |
| Assessment methods | Multiple-choice questions | Case analysis |
| Theoretical basis of the course | Clinical practice guidelines | Clinical medication brochure |
| **What is your choice?** | □ | □ |

**Part Ⅱ：Primary Care Physicians’ Preferences for Training Programs in Prescribing and Deprescribing for Multimorbidity**

This section aims to understand your preferences regarding prescribing and deprescribing training, which will inform the development and optimization of medication regimens for patients with multimorbidity. Your input is crucial for enhancing primary care quality and improving treatment outcomes in this patient population. Completion is estimated to take 2-3 minutes. All responses will be maintained under strict confidentiality.

**（1）The description of attributes and levels**

| **Attribute** | **Definition** | **Level** |
| --- | --- | --- |
| **Instructor composition** | Professionals who offer courses;  Structure of the teaching staff | 1. General practitioners 2. General practitioner   +clinical pharmacist |
| Teaching model | The model that the course is conducted | c)Lectures  d)Lectures+case-based  learning |
| Training location | Whether the training is organized online or on-site | 1. On-site teaching 2. Online teaching |
| Participant enrollment | Enrollment method of participants, i.e., the participants are involved individually or totally | 1. Individual involvement 2. Total involvement |
| Session duration | Time required for a session | 1. 45min 2. 90min |
| Training frequency | How often is each session conducted | 1. Once a month 2. Once a week |
| Assessment methods | The way to measure participant learning outcomes after training, including multiple-choice questions and case analysis | 1. Multiple-choice questions 2. Case analysis |
| Theoretical basis of the course | Main sources of pedagogical content in training courses and materials | 1. Clinical practice guidelines 2. Clinical medication brochure |

**（2）Scenario-based simulation:**

The following section presents 6 choice questions. Each question shares an identical scenario: Suppose you were to receive training on prescribing and deprescribing for patients with multimorbidity. You may choose between two instructional formats that yield identical clinical outcomes but differ in their educational processes. We ask you to consider which format you would prefer.

**The sample question item:**

|  | **Training plan A** | **Training plan B** |
| --- | --- | --- |
| Instructors composition | General practitioner | General practitioner  +Clinical pharmacist |
| Teaching model | Lectures+Case-based learning | Lectures |
| Training location | Online teaching | On-site teaching |
| Participant enrollment | Individual involvement | Total involvement |
| Session duration | 90 min | 45 min |
| Training frequency | Once a week | Once a month |
| Assessment methods | multiple-choice questions | Case analysis |
| Theoretical basis of the course | Clinical practice guidelines | Clinical medication brochure |
| **What is your choice?** | ☑ | □ |

“√” indicates that the respondent selected Training plan A in the sample question. This choice reflects a preference for the specific combination of attribute levels presented in Option A after comparing both alternatives.

In the following six hypothetical scenarios, Training Plan A and Training Plan B are comparable but differ in content. After careful comparison, **please select your preferred plan by marking “✓” in the “□”. Note: There are no correct or incorrect choices**—only differences in training processes, required effort, and time commitment. Please indicate which plan you would prefer.

| **S1** | **Training plan A** | **Training plan B** |
| --- | --- | --- |
| Instructors composition | General practitioner  +clinical pharmacist | General practitioners |
| Teaching model | Lectures+case-based learning | Lectures |
| Training location | Online teaching | On-site teaching |
| Participant enrollment | Individual involvement | Total involvement |
| Session duration | 45min | 90min |
| Training frequency | Once a month | Once a week |
| Assessment methods | Case analysis | Multiple-choice questions |
| Theoretical basis of the course | Clinical practice guidelines | Clinical medication brochure |
| **What is your choice?** | □ | □ |

| **S2** | **Training plan A** | **Training plan B** |
| --- | --- | --- |
| Instructors composition | General practitioner  +clinical pharmacist | General practitioners |
| Teaching model | Lectures+case-based learning | Lectures |
| Training location | On-site teaching | Online teaching |
| Participant enrollment | Total involvement | Individual involvement |
| Session duration | 90min | 45min |
| Training frequency | Once a month | Once a week |
| Assessment methods | Multiple-choice questions | Case analysis |
| Theoretical basis of the course | Clinical practice guidelines | Clinical medication brochure |
| **What is your choice?** | □ | □ |

| **S3** | **Training plan A** | **Training plan B** |
| --- | --- | --- |
| Instructors composition | General practitioner  +clinical pharmacist | General practitioners |
| Teaching model | Lectures | Lectures+case-based learning |
| Training location | On-site teaching | Online teaching |
| Participant enrollment | Total involvement | Individual involvement |
| Session duration | 90min | 45min |
| Training frequency | Once a week | Once a month |
| Assessment methods | Case analysis | Multiple-choice questions |
| Theoretical basis of the course | Clinical practice guidelines | Clinical medication brochure |
| **What is your choice?** | □ | □ |

| **S4** | **Training plan A** | **Training plan B** |
| --- | --- | --- |
| Instructors composition | General practitioners | General practitioner  +clinical pharmacist |
| Teaching model | Lectures | Lectures+case-based learning |
| Training location | Online teaching | On-site teaching |
| Participant enrollment | Total involvement | Individual involvement |
| Session duration | 45min | 90min |
| Training frequency | Once a week | Once a month |
| Assessment methods | Multiple-choice questions | Case analysis |
| Theoretical basis of the course | Clinical practice guidelines | Clinical medication brochure |
| **What is your choice?** | □ | □ |

| **S5** | **Training plan A** | **Training plan B** |
| --- | --- | --- |
| Instructors composition | General practitioner  +clinical pharmacist | General practitioners |
| Teaching model | Lectures+case-based learning | Lectures |
| Training location | On-site teaching | Online teaching |
| Participant enrollment | Individual involvement | Total involvement |
| Session duration | 45min | 90min |
| Training frequency | Once a week | Once a month |
| Assessment methods | Multiple-choice questions | Case analysis |
| Theoretical basis of the course | Clinical medication brochure | Clinical practice guidelines |
| **What is your choice?** | □ | □ |

| **S6** | **Training plan A** | **Training plan B** |
| --- | --- | --- |
| Instructors composition | General practitioners | General practitioner  +clinical pharmacist |
| Teaching model | Lectures+case-based learning | Lectures |
| Training location | Online teaching | On-site teaching |
| Participant enrollment | Total involvement | Individual involvement |
| Session duration | 90min | 45min |
| Training frequency | Once a month | Once a week |
| Assessment methods | Multiple-choice questions | Case analysis |
| Theoretical basis of the course | Clinical medication brochure | Clinical practice guidelines |
| **What is your choice?** | □ | □ |
