# Supplementary material for: Preferences for implementing training program among primary care physicians in prescribing and deprescribing for patients with multimorbidity: a discrete choice experiment
Source: Front Med (Lausanne). 2026 Mar 13;13:1795722. doi: 10.3389/fmed.2026.1795722 (PMC13021398; doi:10.3389/fmed.2026.1795722)
Supplement: Supplementary file 2 [file Supplementary_file_2.docx]

#### Supplementary Appendix: Mixed Logit Model Specification

Data structure: The dataset was organised in long format, with each row representing one alternative (A or B) in a choice task. Variables included id (respondent identifier), case (choice task identifier), choice (dependent variable, 1 if the alternative was chosen, 0 otherwise), and the eight attribute dummy variables.

**Coding of attributes:**

1. instructor_composition:

1 = General practitioner + clinical pharmacist,

0 = General practitioner only.

1. teaching_model:

1 = Lectures + case‑based learning,

0 = Lectures only.

1. training_location:

1 = Online teaching,

0 = On‑site teaching.

1. participant_enrollment:

1 = Total involvement,

0 = Individual involvement.

1. session_duration:

1 = 90 minutes,

0 = 45 minutes.

1. training_frequency:

1 = Once a week,

0 = Once a month.

1. assessment_methods:

1 = Case analysis,

0 = Multiple‑choice questions.

1. theoretical_basis:

1 = Clinical medication brochure,

0 = Clinical practice guidelines.

**Stata command:**

1、mixlogit choice instructor_composition teaching_model training_location ///

participant_enrollment session_duration training_frequency ///

assessment_methods theoretical_basis, ///

group(case) id(id) ///

rand ( instructor_composition teaching_model training_location ///

participant_enrollment session_duration training_frequency ///

assessment_methods theoretical_basis) ///

nrep(500)

**Explanation:** In the mixlogit model, all eight attributes were specified as random parameters using the rand( ) option, each assumed to follow a normal distribution. The model was estimated with 500 Halton draws ( nrep(500) ).

2、clogit choice instructor_composition teaching_model training_location ///

participant_enrollment session_duration training_frequency ///

assessment_methods theoretical_basis, ///

group(case) vce(cluster id)

3、mixlogit choice instructor_composition teaching_model training_location ///

participant_enrollment session_duration training_frequency ///

assessment_methods theoretical_basis, ///

group(case) id(id) ///

rand (instructor_composition participant_enrollment session_duration ///

training_frequency theoretical_basis) ///

nrep(500)

**Explanation:** In the alternative model, the rand( ) option declares the five attributes with significant standard deviations as random parameters, all assumed to follow a normal distribution.

1. gen neg_training_frequency = -training_frequency

gen neg_session_duration = -session_duration

mixlogit choice instructor_composition teaching_model training_location ///

participant_enrollment session_duration training_frequency ///

assessment_methods theoretical_basis, ///

group(case) id(id) ///

rand (instructor_composition teaching_model training_location ///

participant_enrollment assessment_methods theoretical_basis ///

neg_training_frequency neg_session_duration ) ///

ln(2) nrep(500)

**Explanation:** In an additional robustness check, we re‑estimated the mixed logit model assuming that the coefficients for training frequency and session duration follow a log‑normal distribution, while all other random parameters retained the normal distribution.

5、gen workload_high = (daily_patient > 30) if !missing(daily_patient)

label define workload 0 "≤30 patients/day" 1 ">30 patients/day"

label values workload_high workload

gen exp_high = (practice_years > 11) if !missing(practice_years)

label define exp 0 "≤11 years" 1 ">11 years"

label values exp_high exp

mixlogit choice instructor_composition teaching_model training_location ///

participant_enrollment session_duration training_frequency ///

assessment_methods theoretical_basis ///

if workload_high==0， ///

group(case) id(id) ///

rand ( instructor_composition teaching_model training_location ///

participant_enrollment session_duration training_frequency ///

assessment_methods theoretical_basis) ///

nrep(500)

mixlogit choice instructor_composition teaching_model training_location ///

participant_enrollment session_duration training_frequency ///

assessment_methods theoretical_basis ///

if workload_high==1， ///

group(case) id(id) ///

rand ( instructor_composition teaching_model training_location ///

participant_enrollment session_duration training_frequency ///

assessment_methods theoretical_basis) ///

nrep(500)

mixlogit choice instructor_composition teaching_model training_location ///

participant_enrollment session_duration training_frequency ///

assessment_methods theoretical_basis ///

if exp_high==0， ///

group(case) id(id) ///

rand ( instructor_composition teaching_model training_location ///

participant_enrollment session_duration training_frequency ///

assessment_methods theoretical_basis) ///

nrep(500)

mixlogit choice instructor_composition teaching_model training_location ///

participant_enrollment session_duration training_frequency ///

assessment_methods theoretical_basis ///

if exp_high==1， ///

group(case) id(id) ///

rand ( instructor_composition teaching_model training_location ///

participant_enrollment session_duration training_frequency ///

assessment_methods theoretical_basis) ///

nrep(500)

**Explanation:** Each model used the same specification as the main analysis, with all eight attributes included as dummy-coded variables. This approach allowed us to compare coefficient magnitudes across subgroups and examine whether preferences for training attributes differed by workload or professional experience.
