# Supplementary material for: Preferences for implementing training program among primary care physicians in prescribing and deprescribing for patients with multimorbidity: a discrete choice experiment
Source: Front Med (Lausanne). 2026 Mar 13;13:1795722. doi: 10.3389/fmed.2026.1795722 (PMC13021398; doi:10.3389/fmed.2026.1795722)
Supplement: Supplementary file 3 [file Supplementary_file_3.docx]

| **Attributes and Levels** | **β** | **95%CI** | | **SD** | **95%CI** | |
| --- | --- | --- | --- | --- | --- | --- |
|  |  | **Lower** | **Upper** |  | **Lower** | **Upper** |
| Instructors composition (Ref: General practitioner) |  |  |  |  |  |  |
| General practitioner+clinical pharmacist | 0.054 | -0.071 | 0.178 | 0.199 | -0.215 | 0.613 |
| Teaching model (Ref: lectures) |  |  |  |  |  |  |
| Lectures+Case-based learning | -0.078 | -0.194 | 0.039 | 0.003 | -0.201 | 0.207 |
| Training location (Ref: on-site teaching) |  |  |  |  |  |  |
| Online teaching | 0.051 | -0.072 | 0.174 | 0.497* | 0.313 | 0.681 |
| Participant enrollment (Ref: individual involvement) |  |  |  |  |  |  |
| Total involvement | **-0.211*** | -0.327 | -0.094 | 0.017 | -0.469 | 0.503 |
| Session duration (Ref: 45 min) |  |  |  |  |  |  |
| 90 min | **-0.327*** | -0.438 | -0.217 | -0.081 | -0.405 | 0.243 |
| Training frequency (Ref: once a month) |  |  |  |  |  |  |
| Once a week | **-0.345*** | -0.457 | -0.234 | 0.197 | -0.154 | 0.548 |
| Assessment methods (Ref: multi-choice questions) |  |  |  |  |  |  |
| Case analysis | **0.118*** | -0.013 | 0.224 | 0.007 | -0.166 | 0.181 |
| Theoretical basis of the course (Ref: clinical practice guideline) |  |  |  |  |  |  |
| Clinical medication brochure | -0.001 | -0.105 | 0.103 | -0.021 | -0.200 | 0.158 |
| Log-likelihood | -1366.280 | Pseudo-R² | | | 0.046 | |
| AIC | 2766.559 |  | | |  | |
| BIC | 2874.192 |  | | |  | |

****Supplementary Table 1: Mixed logit model estimates of PCPs’ attribute preferences using the full sample****

Footnote: Levels that were statistically significant at 5% level were shown by “ **^*^** ”.

Abbreviation: Ref: reference level. SD: standard deviation. 95% CI: confidence interval.

****Supplementary Table 2: Conditional logit model estimates of PCPs’ attribute preferences****

| **Attributes and Levels** | **β** | **95%CI** | |
| --- | --- | --- | --- |
|  |  | **Lower** | **Upper** |
| Instructors composition (Ref: General practitioner) |  |  |  |
| General practitioner+clinical pharmacist | **0.176*** | 0.014 | 0.338 |
| Teaching model (Ref: lectures) |  |  |  |
| Lectures+Case-based learning | -0.019 | -0.122 | 0.085 |
| Training location (Ref: on-site teaching) |  |  |  |
| Online teaching | 0.000 | -0.162 | 0.162 |
| Participant enrollment (Ref: individual involvement) |  |  |  |
| Total involvement | **-0.150*** | -0.278 | -0.021 |
| Session duration (Ref: 45 min) |  |  |  |
| 90 min | **-0.357*** | -0.492 | -0.221 |
| Training frequency (Ref: once a month) |  |  |  |
| Once a week | **-0.479*** | -0.609 | -0.349 |
| Assessment methods (Ref: multi-choice questions) |  |  |  |
| Case analysis | 0.053 | -0.044 | 0.150 |
| Theoretical basis of the course (Ref: clinical practice guideline) |  |  |  |
| Clinical medication brochure | **-0.113*** | -0.215 | -0.012 |
| Log-likelihood | -956.670 | | |
| AIC | 1931.341 | | |
| BIC | 1985.326 | | |
| Pseudo-R² | 0.072 | | |

Footnote: Levels that were statistically significant at 5% level were shown by “ **^*^** ”.

Abbreviation: Ref: reference level. 95% CI: confidence interval.

****Supplementary Table 3: Mixed logit model estimates of PCPs’ attribute preferences (Five attributes as random parameters)****

| **Attributes and Levels** | **β** | **95%CI** | | **SD** | **95%CI** | |
| --- | --- | --- | --- | --- | --- | --- |
|  |  | **Lower** | **Upper** |  | **Lower** | **Upper** |
| Instructors composition (Ref: General practitioner) |  |  |  |  |  |  |
| General practitioner+clinical pharmacist | **0.217*** | -0.013 | 0.420 | **0.865*** | 0.627 | 1.103 |
| Teaching model (Ref: lectures) |  |  |  |  |  |  |
| Lectures+Case-based learning | -0.004 | -0.182 | 0.175 | - | - | - |
| Training location (Ref: on-site teaching) |  |  |  |  |  |  |
| Online teaching | 0.011 | -0.160 | 0.182 | - | - | - |
| Participant enrollment (Ref: individual involvement) |  |  |  |  |  |  |
| Total involvement | **-0.209*** | -0.386 | -0.032 | 0.083 | -0.419 | 0.585 |
| Session duration (Ref: 45 min) |  |  |  |  |  |  |
| 90 min | **-0.479*** | -0.664 | -0.294 | **0.775*** | 0.529 | 1.020 |
| Training frequency (Ref: once a month) |  |  |  |  |  |  |
| Once a week | **-0.673*** | -0.872 | -0.474 | **0.866*** | 0.629 | 1.113 |
| Assessment methods (Ref: multi-choice questions) |  |  |  |  |  |  |
| Case analysis | 0.056 | -0.094 | 0.205 | - | - | - |
| Theoretical basis of the course (Ref: clinical practice guideline) |  |  |  |  |  |  |
| Clinical medication brochure | **-0.152*** | -0.304 | -0.001 | -0.090 | -0.427 | 0.246 |
| Log-likelihood | -927.431 | Pseudo-R² | | | 0.100 | |
| AIC | 1882.863 |  | | |  | |
| BIC | 1966.839 |  | | |  | |

Footnote: Levels that were statistically significant at 5% level were shown by “ **^*^** ”.

Abbreviation: Ref: reference level. SD: standard deviation. 95% CI: confidence interval.

****Supplementary Table 4: Mixed logit model with log‑normal distribution for training frequency and session duration****

| **Attributes and Levels** | **β** | **95%CI** | | **SD** | **95%CI** | |
| --- | --- | --- | --- | --- | --- | --- |
|  |  | **Lower** | **Upper** |  | **Lower** | **Upper** |
| Instructors composition (Ref: General practitioner) |  |  |  |  |  |  |
| General practitioner+clinical pharmacist | **0.359*** | -0.110 | 0.607 | **0.651*** | 0.322 | 0.980 |
| Teaching model (Ref: lectures) |  |  |  |  |  |  |
| Lectures+Case-based learning | 0.013 | -0.182 | 0.208 | -0.014 | -0.372 | 0.344 |
| Training location (Ref: on-site teaching) |  |  |  |  |  |  |
| Online teaching | -0.044 | -0.303 | 0.215 | **1.312*** | 0.971 | 1.653 |
| Participant enrollment (Ref: individual involvement) |  |  |  |  |  |  |
| Total involvement | **-0.206*** | -0.410 | -0.001 | **0.439*** | 0.054 | 0.823 |
| Session duration (Ref: 45 min) |  |  |  |  |  |  |
| 90 min | **-2.278*** | -4.149 | -0.408 | **2.296*** | 0.944 | 3.649 |
| Training frequency (Ref: once a month) |  |  |  |  |  |  |
| Once a week | **-1.545*** | -2.618 | -0.472 | **2.261*** | 1.214 | 3.307 |
| Assessment methods (Ref: multi-choice questions) |  |  |  |  |  |  |
| Case analysis | 0.078 | -0.093 | 0.250 | 0.199 | -0.470 | 0.867 |
| Theoretical basis of the course (Ref: clinical practice guideline) |  |  |  |  |  |  |
| Clinical medication brochure | **-0.202*** | -0.378 | -0.025 | -0.300 | -0.691 | 0.091 |
| Log-likelihood | -883.998 | Pseudo-R² | | | 0.142 | |
| AIC | 1801.995 |  | | |  | |
| BIC | 1903.967 |  | | |  | |

Footnote: Levels that were statistically significant at 5% level were shown by “ **^*^** ”.

Abbreviation: Ref: reference level. SD: standard deviation. 95% CI: confidence interval.

****Supplementary Table 5: Subgroup analysis by workload: Mixed logit model coefficients for training attributes among PCPs with high vs. low daily patient encounters****

| **Attributes and Levels** | **β-high**  **( >30)** | **95%CI** | | | **β-low**  **(≤30)** | **95%CI** | |
| --- | --- | --- | --- | --- | --- | --- | --- |
|  |  | **Lower** | **Upper** | |  | **Lower** | **Upper** |
| Instructors composition (Ref: General practitioner) |  |  |  | |  |  |  |
| General practitioner+clinical pharmacist | **0.537*** | 0.003 | 1.071 | | 0.315 | 0.006 | 0.636 |
| Teaching model (Ref: lectures) |  |  |  | |  |  |  |
| Lectures+Case-based learning | -0.128 | -0.536 | 0.279 | | 0.139 | -0.125 | 0.402 |
| Training location (Ref: on-site teaching) |  |  |  | |  |  |  |
| Online teaching | -0.073 | -0.544 | 0.398 | | 0.027 | -0.313 | 0.368 |
| Participant enrollment (Ref: individual involvement) |  |  |  | |  |  |  |
| Total involvement | -0.240 | -0.669 | 0.189 | | **-0.351*** | -0.631 | -0.070 |
| Session duration (Ref: 45 min) |  |  |  | |  |  |  |
| 90 min | **-0.748*** | -1.205 | -0.292 | | **-0.426*** | -0.717 | -0.136 |
| Training frequency (Ref: once a month) |  |  |  | |  |  |  |
| Once a week | **-1.160*** | -1.647 | -0.674 | | **-0.547*** | -0.853 | -0.240 |
| Assessment methods (Ref: multi-choice questions) |  |  |  | |  |  |  |
| Case analysis | 0.215 | -0.105 | 0.534 | | 0.030 | -0.199 | 0.259 |
| Theoretical basis of the course (Ref: clinical practice guideline) |  |  |  | |  |  |  |
| Clinical medication brochure | -0.176 | -0.501 | 0.148 | | **-0.219*** | -0.456 | 0.018 |
| Log-likelihood | -383.477 | | | -499.553 | | | |

Footnote: Levels that were statistically significant at 5% level were shown by “ **^*^** ”.

Abbreviation: Ref: reference level. SD: standard deviation. 95% CI: confidence interval.

****Supplementary Table 6: Subgroup analysis by professional experience: Mixed logit model coefficients for training attributes among PCPs with high vs. low years of practice****

| **Attributes and Levels** | **β-high**  **(>11)** | **95%CI** | | **β-low**  **(≤11)** | **95%CI** | |
| --- | --- | --- | --- | --- | --- | --- |
|  |  | **Lower** | **Upper** |  | **Lower** | **Upper** |
| Instructors composition (Ref: General practitioner) |  |  |  |  |  |  |
| General practitioner+clinical pharmacist | **0.792*** | 0.227 | 1.356 | 0.280 | -0.048 | 0.608 |
| Teaching model (Ref: lectures) |  |  |  |  |  |  |
| Lectures+Case-based learning | 0.409 | -0.027 | 0.845 | -0.100 | -0.387 | 0.187 |
| Training location (Ref: on-site teaching) |  |  |  |  |  |  |
| Online teaching | -0.377 | -0.850 | 0.096 | 0.355 | -0.013 | 0.724 |
| Participant enrollment (Ref: individual involvement) |  |  |  |  |  |  |
| Total involvement | -0.438 | -0.885 | 0.009 | -0.202 | -0.502 | 0.099 |
| Session duration (Ref: 45 min) |  |  |  |  |  |  |
| 90 min | **-0.601*** | -1.006 | -0.197 | **-0.628*** | -0.948 | -0.308 |
| Training frequency (Ref: once a month) |  |  |  |  |  |  |
| Once a week | **-1.326*** | -1.882 | -0.769 | **-0.617*** | -0.978 | -0.256 |
| Assessment methods (Ref: multi-choice questions) |  |  |  |  |  |  |
| Case analysis | 0.079 | -0.260 | 0.418 | 0.146 | -0.093 | 0.385 |
| Theoretical basis of the course (Ref: clinical practice guideline) |  |  |  |  |  |  |
| Clinical medication brochure | -0.320 | -0.644 | 0.003 | -0.091 | -0.339 | 0.157 |
| Log-likelihood | -437.909 | | | -450.986 | | |

Footnote: Levels that were statistically significant at 5% level were shown by “ **^*^** ”.

Abbreviation: Ref: reference level. SD: standard deviation. 95% CI: confidence interval.
